# Sleep Disorders in Autism Spectrum Disorder Pre-School Children: An Evaluation Using the Sleep Disturbance Scale for Children

**DOI:** 10.3390/medicina57020095

**Published:** 2021-01-22

**Authors:** Domenico M. Romeo, Claudia Brogna, Arianna Belli, Simona Lucibello, Costanza Cutrona, Massimo Apicella, Eugenio Mercuri, Paolo Mariotti

**Affiliations:** 1 Pediatric Neurology Unit, Fondazione Policlinico Universitario A. Gemelli, IRCCS, 00168 Rome, Italy; eugeniomercuri@unicatt.it (E.M.); paolo.mariotti@policlinicogemelli.it (P.M.); 2 Pediatric Neurology Unit, Università Cattolica del Sacro Cuore, 00168 Rome, Italy; claudiabrogna@yahoo.it (C.B.); arianna.belli3@gmail.it (A.B.); simonalucibello@tiscali.it (S.L.); cutrona.co@gmail.com (C.C.); drmassimoapicella@gmail.com (M.A.); 3 Neuropsichiatria Infantile, ASL Avellino, 83100 Avellino, Italy

**Keywords:** autism, sleep disorders, pre-school children

## Abstract

*Background and Objectives:* Sleep disorders are common in children with Autism Spectrum Disorder (ASD). The aims of this study were to describe the incidence and characteristics of sleep disorders using a questionnaire completed by the caregiver in a sample of preschool-aged children with ASD and to identify possible differences in a control group of peers. *Materials and Methods:* Sleep disorders were investigated with the Sleep Disturbance Scale for Children (SDSC) in a population of pre-school-aged (3–5 years) ASD children and in a control group. The Autism Diagnostic Observation Schedule—second ed. (ADOS-2) was further used to assess autism symptom severity. A total of 84 children (69 males; mean age 3.9 ± 0.8 years) with a diagnosis of ASD and 84 healthy controls (65 males; mean age of 3.7 ± 0.8 years) that were matched for age and sex were enrolled. *Results:* ASD children reported significantly higher (pathological) scores than the control group on the SDSC total scores and in some of the factor scores, such as Difficulty in Initiating and Maintaining Sleep (DIMS), disorders of excessive somnolence (DOES), and sleep hyperhidrosis. A total of 18% of ASD children had a pathological SDSC total T-score, and 46% had an abnormal score on at least one sleep factor; DIMS, parasomnias, and DOES showed the highest rates among the sleep factors. Younger children (3 years) reported higher scores in DIMS and sleep hyperhidrosis than older ones (4 and 5 years). No specific correlation was found between ADOS-2 and SDSC scores. *Conclusions:* Pre-school children with ASD showed a high incidence of sleep disorders with different distributions of specific sleep factors according to their age. We suggest a screening assessment of sleep disorders using the SDSC in these children with a more in-depth evaluation for those reporting pathological scores on the questionnaire.

## 1. Introduction

Autism spectrum disorders (ASDs) are a heterogeneous group of neurodevelopmental conditions affecting an individual’s communication, social interaction, and behavior, with symptoms that are not better explained by an intellectual disability or a global developmental delay. The disturbances caused by ASD are present starting in the early developmental period and cause clinically significant impairment in important areas of a child’s functioning [1].

Among the medical conditions known to be associated with ASD, sleep disorders stand out as one of the most commonly reported; their prevalence in ASD children range from 45% up to 86% [2,3,4,5,6,7,8], and they have been shown to have a more chronic nature than those affecting typically developing children [6,7], thus having a significant impact on the health and quality of life of both patients and their caregivers. It has indeed been found that autistic chidren with sleep disorders experience increased daytime symptoms of anxiety, depression, aggressiveness, and attention deficit, as well as greater social impairment and poorer perfomances in cognitive and sensory–motor tasks, compared to autistic children who do not have sleep problems [9,10,11]. 

A sleep assessment should, therefore, be performed systematically in children with ASD as soon as possible for an early and specific treatment that could improve not only the sleep quality, but also the related behavioral disorders. Although objective measures of sleep, such as actigraphy and polysomnography, could provide more accurate information for diagnosis and treatment planning, subjective measures of sleep, such as parent-report questionnaires, appear to be the most appropriate for screening purposes, considering their tolerability for the patients and caregivers, their wide availability, and the limited cost, time, and training needed for their administration and scoring [12,13].

Several standardized scales are available for identifying sleep disorders in childhood, but a recent review [12] showed that only a few of them fulfill all the methodological steps needed to adequately develop and evaluate a sleep assessment tool. Among the questionnaires that meet these requirements, the Sleep Disturbance Scale for Children (SDSC) [13,14] has been widely used in cohorts of children with various medical conditions, such as attention deficit and hyperactivity disorder, cerebral palsy, epilepsy, and genetic syndromes [15,16,17]. However one study only used the SDSC in school-age children with ASD, confirming higher sleep problems in this population of infants [18], but no study used the SDSC version specifically validated for pre-school age in this population of children.

The aims of this study were (i) to describe the incidence and characteristics of sleep disorders using the SDSC in a sample of preschool-aged children with ASD and (ii) to identify possible differences in a control group of peers. We expected to observe a higher incidence of sleep disorders in children with ASD than in the control group, even in a population of pre-schoolers. 

## 2. Materials and Methods

### 2.1. Participants

The children included in this study were collected retrospectively using clinical records of pre-school children with a diagnosis of ASD who were regularly followed at the Child Neurology Unit of the Catholic University of Rome between January 2018 and December 2019. 

Children in the study group met the following inclusion criteria: (a) age range from 3 to 5 years, (b) met the criteria for ASD on the Diagnostic and Statistical Manual of Mental Disorders—DSM-V [1] according to clinical judgment by clinicians with diagnostic expertise, and (c) met the criteria for ASD on the Autism Diagnostic Observation Schedule—second edition (ADOS-2) [19].

Patients with known genetic or chromosomal abnormalities, neuromotor disorders, significative sensorial impairment (profund bilateral sensory neural hearing loss, profound bilateral vision impairment), a diagnosis of epilepsy, or active antiepileptic drugs treatment were excluded from the study, as these conditions might have an independent impact on sleep quality. 

A group of healthy children was used as a control group; they all attended regular classes in mainstream nursery school, and those with obvious or reported sign of mental, developmental, or physical disabilities, according to school medical records or reception of on-going prescription medication (antiepileptic drugs, antihistaminic drugs, benzodiazepine, melatonin), were excluded.

The study was approved by the Research Ethics Board of our institution (ID: 3418, prot. N. 0037324/20). Informed consent was obtained from parents of the children included in the present study.

### 2.2. Sleep Disturbance Measure

Sleep disturbances were assessed using the Sleep Disturbance Scale for Children (SDSC) validated for pre-school children [13]. This questionnaire was distributed consecutively during the study period to the primary caregivers of pre-school children with a diagnosis of ASD, during the routine clinical assessment in our unit, and under the supervision of the researchers (authors: C.B., A.B., and C.C.).

The SDSC is an evaluation tool specifically developed for pediatric ages that investigates the occurrence of sleep disorders in the previous six months. It consists of 26 items in a five-point Lykert-type scale (1 = least severe and 5 = most severe). The sum of the single items’ scores provides a total sleep score that possibly ranges from 26 to 130. A total sleep score of >70 is considered pathological. The SDSC was chosen due to its psychometric properties: The internal consistency is good in spite of the relative heterogeneity of the items; both item and total scores do not change when tested and retested; furthermore, the six factors represent the most common areas of sleep disorders in childhood.

The SDSC was originally validated on a sample of typically developed and healthy children aged 6 to 16 years [14], and was then validated for preschool-aged children as well [13]. In this age range (3 to 6 years old), a different factorial structure from the original SDSC was found due to a different prevalence of sleep disturbances in younger children, although the cut-off for the total SDSC score remained similar. The most common areas of sleep disturbance in preschoolers were divided into six factors: parasomnias (PAR), difficulty in initiating and maintaining sleep (DIMS), sleep-disordered breathing (SDB), disorders of excessive somnolence (DOES), sleep hyperhidrosis (SHY), and non-restorative sleep (NRS) [13]. For the present study, the primary caregiver completed the pre-school version of the SDSC.

The SDSC was further distributed to the primary caregivers of a group of healthy children recruited via nurseries and considered as a control group. Questionnaires were filled out by the caregiver during the school hours under the supervision of the researchers that distributed the questionnaires (authors: C.B., A.B., and C.C.); no missing values were reported. 

### 2.3. Autism Symptom Severity Measure

The severity of autistic symptoms of the pre-school children with a diagnosis of ASD was evaluated by trained examiners (authors: S.L. and P.P.) using the ADOS-2 [19]. This test has a good diagnostic accuracy and interperson objectivity [20], and it is considered the gold standard for diagnosing ASD. It consists of a standardized and semi-structured evaluation of communication, social interaction, play, and imagination, and is designed for the assessment of children and adults presenting clinical features suggestive of ASD. It has five different modules consisting of different activities in order to observe the behavior of participants with various developmental and language levels. Each patient is assessed with only one module. For the purposes of our study, modules 1, 2, and 3 were used (module 1 for minimally verbal patients; module 2 for patients with phrase speech; module 3 for individuals with fluent speech).

The activities of the ADOS-2 were proposed by a trained examiner to the patient in order to observe the patient’s behavior; the observed behavior was coded on a four-point scale, with code 0 being “when the behavior shows no evidence of abnormality as specified” and code 3 being “when the behavior is markedly abnormal in a way that interferes with the interview”.

A selection of the scores attributed to the behavioral observations was then entered into an algorithm, resulting in two scores (“social affection” score, AS, and “restricted and repetitive behaviors” score, RRB). The sum of these two scores gave a total score, which matched with a comparison score (also known as calibrated severity score, CSS), from 1 to 10, indicating the severity of the autistic symptoms (i.e., little-to-no, mild-to-moderate, and moderate-to-severe symptoms). All modules included two cut-off scores: a lower cut-off score for the classification of “autism spectrum disorder” and a higher cut-off score for the classification of “autism”.

The researchers conducted a blind examination of the sleep questionnaire data and ADOS scores at the time of the analysis.

### 2.4. Data Analysis

Data were presented as mean values and standard deviations for continuous variables and as counts and percentages for categorical variables. 

The comparisons between children with ASD and the control group for the SDSC total and the six-factor scores and ages were performed using the non-parametric Mann–Whitney U test, as the data were not normally distributed; the comparisons for the gender was performed with Fisher’s exact test. 

Children were subdivided into three groups according to their age at the time of assessment (3, 4, or 5 years). Analysis of variance (ANOVA) was used to define possible differences of ADOS-2 and SDSC scores according to the three ages of assessments followed by a post-hoc analysis for multiple comparisons using Bonferroni’s method. The non-parametric Wilcoxon signed-rank test was used to compare differences according to SDSC total and factor scores. The correlation between the ADOS-2 total score, AS, RRB and CSS, and total SDSC and SDSC factor scores were explored using the Spearman test. A two-tailed value of *p* < 0.05 was considered significant.

## 3. Results

During the study period, 84 children with ASD (69 males, 15 females) and their primary caregivers, which were their mothers in all cases, fulfilled the inclusion criteria. The mean age was 3.9 ± 0.8 years (range: 3–5 years). A total of 40 children were aged 3 years (3 years to 3 years and 11 months), 26 were aged 4 years (4 years to 4 years and 11 months), and 18 were aged 5 years (5 years to 5 years and 11 months). 

The questionnaire was also completed by the mothers of 84 healthy children (65 males, 19 females) with a mean age of 3.7 ± 0.8 years (range: 3–5 years). A total of 39 children were aged 3 years (3 years to 3 years and 11 months), 25 were aged 4 years (4 years to 4 years and 11 months), and 20 were aged 5 years (5 years to 5 years and 11 months). This control group presented the same distribution (*p* > 0.05) of age and gender compared to the study group. 

### 3.1. SDSC Results

An abnormal total sleep score (>70) was found in 15 (18%) children with ASD, and 39 (46%) had an abnormal score on at least one SDSC factor: PAR 11%, DIMS 22%, SDB 6%, DOES 13%, SHY 12%, and NRS 13%. The SDSC total and factor scores are reported in Table 1. DIMS, PAR, and DOES reported the higher mean scores among ASD children.

ASD children reported no significant differences (*p* > 0.05) between the three age groups (3, 4, and 5 years) in the SDSC total and factor scores, except for the DIMS (*p* = 0.009) and SHY scores (*p* = 0.030), which had higher scores in the group of 3 years (Table 2). No gender differences (*p* > 0.05) were observed in the SDSC total or factor scores.

In the control group, an abnormal SDSC total score was found in four children (5%), and 13 (15%) reported an abnormal score on at least one SDSC factor. No gender or age differences (*p* > 0.05) were observed in the SDSC total or factor scores.

The ASD children reported significantly higher scores than the control group for the SDSC total (*p* = 0.0001), DIMS (*p* = 0.009), DOES (*p* = 0.010), and SHY (*p* = 0.003) scores, whereas no statistical differences (*p* > 0.05) were found for PAR, SDB, and NRS scores (Table 1).

### 3.2. ADOS-2 Results

The mean total ADOS-2 score was 17.7 ± 5.2, the mean AS score was 12.8 ± 4.7, the mean RRB score was 4.9 ± 2, and the mean CSS score was 6.8 ± 1.9. No significant differences were found between the three age groups (3, 4, and 5 years) in the ADOS total, AS, RRB, or CSS scores (Table 2). 

No significant correlation (*p* > 0.05) was found between the ADOS-2 scores (total, AS, RRB, and CSS) and the SDSC total or factor scores. 

## 4. Discussion

Sleep disturbances are common in children with ASD [2,3,4,5,6,7,8,9,10,11,21,22], even if no consensus has been reached about the prevalence of this disorder, probably due to the different tools used as subjective (questionnaires, sleep diaries) or objective assessments (actigraphy, polysomnography) or due to the inclusion of children across wide age ranges. Very few studies have involved young children at pre-school age [2,5,23,24] or considered the associations between the severity of autism symptoms and sleep problems [23]. The possibility of identifying sleep problems early in ASD pre-school children is an important issue, as sleep disturbances often arise during early childhood, making them a first warning sign for parents long before a diagnosis is made [25].

In the present study, the SDSC was used for the first time to assess the sleep disorders in a narrow age range—thus reducing possible age-related changes—observed in both typical and ASD pre-school children. 

Our findings showed that, at pre-school age, children with ASD reported significantly higher scores than the control group on SDSC scores, with 46% of them presenting at least one sleep disorder compared to only 15% only of the control group. These data are in line with the literature reporting differences in sleep behavior problems between young children with ASD (occurring between 40–80%) and typically developing children of similar ages (9–40%) [24,25,26,27,28]. The high prevalence of sleep disorders in preschool ASD is probably a result of multiple interactions between the presence of biological (melatonin metabolism and developmental disruption of other neurotransmitter systems critical to sleep), behavioral/psychological, disrupted sleep architecture, and environmental factors [9,28]. Irregularity in melatonin levels and secretion patterns and abnormalities in clock-related genes have been suggested as potential intrinsic factors, especially in pre-pubertal ages [5,9,23].

In our sample, the ASD children reported higher scores than the control group in SDSC total scores and in most of the six factors. Considering the SDSC factors, DIMS, PAR, and DOES had the highest pathological scores in ASD children and were significantly higher than in the control group. 

Our findings are further consistent with the literature on children and adolescents with ASD, which highlights overall specific sleep disorders, such as DIMS, with more bedtime resistance, sleep-onset delay and latency, sleep anxiety, night awakenings, parasomnias, and a significantly lower sleep duration [27,29,30]. In another study using the same tool but at older ages, ASD children reported higher SDSC scores than the control group. The most common sleep problems reported in children with ASD were sleep–wake transition disorders, followed by disorders of initiation and maintenance [18]. 

Previous research [31] underlined that children with ASD appear to have difficulty transitioning from stimulating activities to sleep, or they may have sensitivities to the level of light or sound in their bedroom, resulting in difficulty falling asleep at night and sleepiness during the following day. 

Sleep symptom profiles could change with age, tracking common problems observed in similarly aged typically developing children [32]. In children with ASD, the sleep problems could fluctuate with age as their symptomology either increases or decreases [5]. In our study, no significant differences in SDSC total or in most factor scores were reported according to the age of assessment (3, 4, or 5 years), probably due to the narrow age range of the sample. The DIMS and SHY factors only were significantly higher in ASD children at younger ages. DIMS had frequently abnormal scores in healthy children at 3–5 years [9,16], but it was even more frequent in the ASD group, as it had a more general association with behavioral dysregulation and may reflect parents’ bedtime management strategies, too [23]; caregivers of ASD preschool children probably tend to pay more attention to making sure that their children get enough sleep than caregivers of children without ASD [28], and in the present study, this seems to be more pronounced for younger children. 

A high prevalence of PAR was also evidenced in our population of pre-school children with ASD, as previously reported in a systematic review of youth with ASD [29]; PAR includes arousal disorders (sleepwalking, sleep terrors), nightmares, and sleep–wake transition disorder; in our study, the high incidence of SHY, especially at younger ages, could be correlated with the higher rate of PAR in ASD children than the control group for the item related to nocturnal hyperkinesia and to the autonomic dysregulation of the neural control of the skin [25], which could lead to increased sweating for the night sweating item.

In the present study, we did not find any significant correlations between ADOS-2 scores and SDSC total or factor scores. Although sleep disorders are recognized as correlating with anxiety, depression, and attention and aggression problems, a direct correlation with autism core features is debated [23,33], and a reciprocal influence can be explained as epiphenomena of a common neurobiological basis, which may reside in altered neurotransmitter metabolism [34], clock gene expression, or melatonin metabolism [35]. So far, recent studies in the literature have reported that the etiology of sleep problems in ASD is multi-factorial; although they are part of the construct of ASD, they do not predict the severity of autistic traits over time, and probably represent a condition not correlated with autism symptoms, but related to associated psychiatric comorbidities in individuals with ASD [23,33,36]. 

A limitation of this study may be found in the lack of an objective measure, such as a structured in-depth interview and objective assessment, which may have provided better information on sleep disorders and allowed a more accurate diagnosis [37]. However, actigraphy and polysomnography in young children may be poorly tolerated, may increase bed anxiety, and are not always feasible in everyday clinical practice; on the other hand, parental questionnaires like the SDSC have the advantage of saving time and costs, can be administered at each outpatient assessment, and can measure a wide range of sleep parameters in various contexts [12,13]. 

Another limitation of this study is that the effect on the main outcome of the intelligence quotient was not assessed. However, the degree of cognitive impairment does not seem to influence the prevalence of sleep problems in ASD, as they are observed in those with a severe intellectual disability as well as those who are high functioning with intelligence quotients greater than 70 [38]. 

A further limitation is that our population was quite predominantly male, and these data could be considered as having a potential selection bias. However, the male/female prevalence in our population is similar to that reported in the literature, as males are about four times more likely to be diagnosed with ASD than females (40), and therefore, this prevalence should be considered a typical finding when assessing ASD children.

## 5. Conclusions

In conclusion, this study points out the high incidence of sleep disorders in ASD children even at pre-school age and that these disorders are poorly related to the severity of autism symptoms. Sleep disorders in pre-school ASD children should be actively explored using a questionnaire like the SDSC in order to identify children that need a more detailed assessment for a conclusive diagnosis and to implement early behavioral strategies [39] and/or specific treatments [40] that might lead to an improvement in behavioral problems, thus increasing the compliance of children with educational interventions.

## Figures and Tables

**Table 1:** Sleep Disturbance Scale for Children (SDSC) total and factor scores in the autism spectrum disorder (ASD) group and the control group.

	PAR	DIMS	SBD	DOES	SHY	NRS	SDSC Total Score
ASD (*n* = 84) Mean and SD	55.2 ± 11.9	60.5 ± 16.3 *	50.3 ± 10.6	55.6 ± 13.3 *	54.1 ± 13.8 *	52.4 ± 14.6	58.8 ± 13.2 *
Control group (*n* = 84) Mean and SD	53.2 ± 10.4	54.8 ± 11.2	50.4 ± 8.4	51.1 ± 8.5	48.9 ± 7.3	52.7 ± 9.8	51.9 ± 9.9

* *p* < 0.05; ASD = autism spectrum disorder; PAR = parasomnias; DIMS = difficulty in initiating and maintaining sleep; SDB = sleep-disordered breathing; DOES = disorders of excessive somnolence; SHY = sleep hyperhidrosis; NRS = non-restorative sleep.

**Table 2:** SDSC total and factor scores and Autism Diagnostic Observation Schedule—second ed. (ADOS-2) total scores according to the age.

	SDSC							ADOS 2			
Age Groups	PAR	DIMS	SBD	DOES	SHY	NRS	Total Score	Total Score	AS Score	RRB Score	CCS Score
3 Years (N = 40) Mean and SD	55.2 ± 11.5	65.4 ± 16.7 *	49.4 ± 8.7	57.3 ± 15.3	57.7 ± 12.4 *	54.6 ± 15.8	61.4 ± 14.0	17.9 ± 5.2	13.1 ± 4.7	4.9 ± 1.9	6.7 ± 1.9
4 Years (N = 26) Mean and SD	54.4 ± 10.1	57.0 ± 12.2	51.3 ± 11.2	55.8 ± 11.2	52.7 ± 16.5	48.7 ± 13.0	57.1 ± 11.0	18.6 ± 5.5	13.5 ± 4.7	5.0 ± 2.7	7.3 ± 1.8
5 Years (N = 18) Mean and SD	56.0 ± 15.4	54.6 ± 18.0	50.9 ± 13.6	51.6 ± 11.0	48.3 ± 10.3	52.8 ± 13.7	55.6 ± 13.8	15.6 ± 4.4	11.1 ± 4.6	4.5 ± 1.6	6.1 ± 1.5

* *p* < 0.05; PAR = parasomnias; DIMS = difficulty in initiating and maintaining sleep; SDB = sleep-disordered breathing; DOES = disorders of excessive somnolence; SHY = sleep hyperhidrosis; NRS = non-restorative sleep.

## Data Availability

Data available on request due to privacy. The data presented in this study are available on request from the corresponding author. The data are not publicly available due to privacy.
